# Bioprinting of Decellularized Porcine Cardiac Tissue for Large-Scale Aortic Models

**DOI:** 10.3389/fbioe.2022.855186

**Published:** 2022-03-10

**Authors:** Beu P. Oropeza, Jason R. Adams, Michael E. Furth, Jack Chessa, Thomas Boland

**Affiliations:** ^1^ Biomedical Device, Delivery and Diagnostic Laboratory, Metallurgical, Materials and Biomedical Engineering Department, The University of Texas at El Paso, El Paso, TX, United States; ^2^ Aerospace and Mechanical Engineering, The University of Texas at El Paso, El Paso, TX, United States

**Keywords:** decellularization, tissue engineering and regenerative medicine, bioprinting, biomechanics, biomaterials, extracellular matrix

## Abstract

Bioprinting is an emerging technique used to layer extrudable materials and cells into simple constructs to engineer tissue or arrive at *in vitro* organ models. Although many examples of bioprinted tissues exist, many lack the biochemical complexity found in the native extracellular matrix. Therefore, the resulting tissues may be less competent than native tissues—this can be especially problematic for tissues that need strong mechanical properties, such as cardiac or those found in the great vessels. Decellularization of native tissues combined with processing for bioprinting may improve the cellular environment for proliferation, biochemical signaling, and improved mechanical characteristics for better outcomes. Whole porcine hearts were decellularized using a series of detergents, followed by lyophilization and mechanical grinding in order to produce a fine powder. Temperature-controlled enzymatic digestion was done to allow for the resuspension of the decellularized extracellular matrix into a pre-gel solution. Using a commercial extrusion bioprinter with a temperature-controlled printhead, a 1:1 scale model of a human ascending aorta and dog bone shaped structures were printed into a reservoir of alginate and xanthium gum then allowed to crosslink at 37C. The bioengineered aortic construct was monitored for cell adhesion, survival, and proliferation through fluorescent microscopy. The dog bone structure was subjected to tensile mechanical testing in order to determine structural and mechanical patterns for comparison to native tissue structures. The stability of the engineered structure was maintained throughout the printing process, allowing for a final structure that upheld the dimensions of the original Computer-Aided Design model. The decellularized ECM (Ē = 920 kPa) exhibited almost three times greater elasticity than the porcine cardiac tissue (Ē = 330 kPa). Similarly, the porcine cardiac tissue displayed two times the deformation than that of the printed decellularized ECM. Cell proliferation and attachment were observed during the *in vitro* cell survivability assessment of human aortic smooth muscle cells within the extracellular matrix, along with no morphological abnormalities to the cell structure. These observations allow us to report the ability to bioprint mechanically stable, cell-laden structures that serve as a bridge in the current knowledge gap, which could lead to future work involving complex, large-scale tissue models.

## Introduction

In the United States alone, 40,000 children are born with a congenital heart problem each year (Jenkins et al., 2007). The current procedures used to treat aortic defects involve synthetic materials, usually textiles, as grafts that replace aortic sections or stents that give additional support to the aortic wall (Kleigman, 2011). Each of these options results in numerous surgical interventions throughout a child’s developing years, leading to extended hospital stays, and potential trauma (Fawzy et al., 2004; Walhout et al., 2004; Carr et al., 2005). Additionally, it has been found that children who suffer from congenital heart defects can be subject to developmental and language delays (Limperopoulos et al., 2002; Easson et al., 2019; Fourdain et al., 2019). Bioprinting, an emerging technique in the biomedical engineering field, has the potential to solve the problems that arise from repetitive surgical intervention. Bioengineers are able to extrusion print using materials specifically characterized for the engineered tissue. The layered biomaterials and cells form precise structures modeled from native biological tissues that can grow along with the child (Murphy and Atala, 2014; Zhang et al., 2016; Rider et al., 2018).

Although many tissues have been engineered using bioprinting, maintaining the biochemical complexity and structural integrity necessary to retain native function has posed a problem in the past. The decellularization of native tissues in order to acquire the extracellular matrix (ECM) as a whole or as components has allowed researchers to bridge the gap in biochemical mimicry. It has been found that ECM helps control tissue-specific functions using interactions with the surrounding cells through a dynamic bidirectional system extending past the cell membrane to the level of gene expression (Bissell et al., 1982; Bornstein et al., 1982; Nelson and Bissell, 2006). This relationship between the cell and its surroundings stimulates proliferation, cell growth and, through the presence of laminin and fibrin, increases cell adhesion properties (Ott et al., 2008; Badylak et al., 2009; Park and Woo, 2012; Wolf et al., 2012).

Equal importance must be given to the mechanical properties of the engineered tissues. As with all living things, these structures are meant to exist in a dynamic environment, oftentimes with relatively large forces acting upon them (Fung, 1933). Removing the tissue from its native environment and processing it for decellularization leads to alterations in the ECM’s physical, structural, and mechanical characteristics (Gilbert et al., 2006). This study places particular interest in defining the ECM’s characteristics prior to decellularization as well as after the bioprinting process is complete. Using samples of porcine cardiac tissue as a control, we compare it to printed dECM and assess the physiological and biochemical makeup, surface topography, and mechanical characteristics.

## Materials and Methods

### Decellularization of Porcine Hearts

Fresh whole porcine hearts were acquired from Nebraska Scientific (Omaha, NE) and cut down to sections not exceeding 2 mm^3^. The tissues were then placed in 2.5 L of a 1% SDS solution for 48 h, stirred continuously. The solution was replaced at 24 h, and it was ensured no agglomerations had formed. The cardiac tissue was then treated with 1% Triton X-100 for 30 min followed by 3 days of PBS washes, where the buffer was replaced every day, and the tissues are stirred continuously. To sterilize the decellularized tissue, it was treated with 1% peracetic acid for 4 h and washed five times with sterile PBS and sterile distilled water. All decellularization steps were done at room temperature. The strained tissue was then frozen at -80°C overnight and lyophilized. All decellularization techniques followed were taken from Pati *et al.* with minimal modification (Pati et al., 2014).

### Cell Culture

Human Aortic Smooth Muscle Cells were grown in vascular growth media (ATCC PCS-100-030) plus smooth muscle kit (ATCC PCS-100-042) under normal environmental culture conditions at 37°C with 5% CO_2_ in a fully aseptic environment. Before printing, cell membranes were stained using PKH26 red fluorescent live cell membrane stain (Sigma MINI26-1KT).

### Bioprinting

The lyophilized dECM was digested and re-suspended in order to form a printable gel material. Lyophilized dECM was mechanically pulverized using burr grinding methods, then 3 g of dECM were digested with 30 mg of pepsin from porcine gastric mucosa (Sigma P7125-100G, ≥3,200 units/mg protein) in 100 ml of 0.5 M acetic acid for 48 h at room temperature with continuous stirring (Pati et al., 2014). Centrifugation at 500 x *g* for 10 min prior to pH adjustment was done to allow separation of any particles that were not solubilized; this allowed for a homogenous texture when printing. Ice cold 10M NaOH was used to adjust the pH of the dECM solution to the physiological range; while the dECM was kept below 10°C to avoid premature crosslinking of the solution. A Bio X bioprinter (Cellink, Boston, MA) equipped with a cooling printhead and an 18-gauge blunt needle was used for all printed samples. Printhead temperature was maintained at 14°C to avoid premature crosslinking of the bioink, print bed temperature was kept at 30°C to help begin the crosslinking process prior to the 1-hour incubation period to achieve full gelation. A print stabilizing solution of 1% Xanthium Gum was used to ensure the stability of the print prior to gelation, a modification of the technique described in Noor et al. (2019). For the cell-laden bio printed structure a cell suspension of 1 × 10 (Limperopoulos et al., 2002) cells/mL was mixed with the solubilized, pH-adjusted dECM immediately prior to bioprinting. Printhead and print bed temperatures were maintained at 14 and 30°C, respectively.

Autodesk Fusion 360 was used for Computer-Aided Design (CAD). The tensile testing shape was drawn to ASTM D412- Die A for thermoplastic elastomers, with minor modifications due to the biological nature of the samples. Dimensions for the aorta construct were taken from previous literature (Walther et al., 1985; Jarvisalo et al., 2001; Skilton et al., 2005; Taketazu et al., 2017).

### Histological Staining

The tissue samples were processed using a Thermo Scientific Spin Tissue Processor Microm STP-120. Tissues were fixed using 4% formalin at 4°C overnight, followed by dehydration. Samples were dehydrated in increasing ethanol concentrations (starting with 70, 95, and 100%), followed by immersion in xylene two times and paraffin infiltration. Paraffin-embedded tissues were sectioned at 4–6 μm using a Shandon Finesse ^®^ E/ME microtome. All samples were deparaffinized in three xylene washes then rehydrated in decreasing ethanol concentrations (100, 95, 70, and 50%), with each wash lasting 3 min. Hematoxylin and eosin (H&E) staining was done post-deparaffinization. Following rehydration, the samples were exposed to hematoxylin solution for 2 min, then rinsed with running water for 1-minute, distilled water for 30 s, and 95% alcohol for an additional 30 s. Immediately after, the samples were exposed to eosin solution for 1 min, then washed with increasing amounts of alcohol (95% x2, 100% x2) for 2 min each, then two xylene washes each for 2 min. Trichrome staining was done using Masson’s Trichrome Staining Kit; all kit procedures were precisely followed. All samples were mounted using Cytoseal™60 (Thermo Scientific REF#8310-4).

### Microscopy

For scanning electron microscopy imaging, samples were fixed using 4% formalin and dehydrated in increasing ethanol concentrations prior to lyophilization. All samples were sputter-coated for 20 s and viewed using a Hitachi S-4800-II SEM. A Nikon AZ100 inverted light microscope was used for histological imaging.

### Mechanical Testing

Uniaxial tensile testing was done at room temperature in order to determine the mechanical properties of native tissue and decellularized printed tissue. One group of samples was taken from a fresh whole porcine heart; rectangles were cut vertically along the sagittal plane. Each sample’s thickness was determined by measurements using a digital caliper. The second sample group consisted of printed decellularized ECM made to ASTM standard D638—Type I and also measured using a digital caliper. All specimens were patted dry, fixed to grips, and placed in the uniaxial electromechanical tensile testing machine (MTESTQuattro), where they were subjected to a strain rate of 5 mm min^−1^ at room temperature until rupture. Forces were measured by a 200 lb. load cell and elongation by the internal sensor as well as by displacement of the reference grid.

### Tensile Data Analysis

The stress-strain relationships for the samples tested were obtained using the Mooney-Rivlin Model for incompressible hyperelastic materials (Peattie et al., 1996; Rubin and Krempl, 2010; Ruiz de Galarreta et al., 2016). Where:

$$Tensile\;stress\;\sigma =\frac{F}{A_{o}}$$ (1)

$$Stretch\;ratio\;\lambda =\frac{\Delta L}{L_{0}}-1$$ (2)

Because the mechanical testing conducted was a uniaxial tensile test:

$$\sigma_{1\;1}-\sigma_{3\;3}=2C_{1}\left(\lambda^{2}-\frac{1}{\lambda }\right)-2C_{2}\left(\frac{1}{\lambda^{2}}-\lambda \right)$$ (3)

And:

$$\sigma_{2\;2}-\sigma_{3\;3}=0$$ (4)

In this case, the model can be further simplified under the assumption of simple tension making:

$$\sigma_{2\;2}=\sigma_{3\;3}=0$$ (5)

Therefore:

$$\sigma_{1\;1}=\left(2C_{1}+\frac{2C_{2}}{\lambda }\right)\left(\lambda^{2}-\frac{1}{\lambda }\right)$$ (6)

This data was then plotted to find the predicted stress-stretch ratio diagrams. Young’s modulus was derived from the slope at the linear region of the graph. Poisson’s ratio was determined by taking the transversal elongation and dividing by axial compression.

## Results

### Decellularization of Porcine Hearts

**FIGURE 1:**
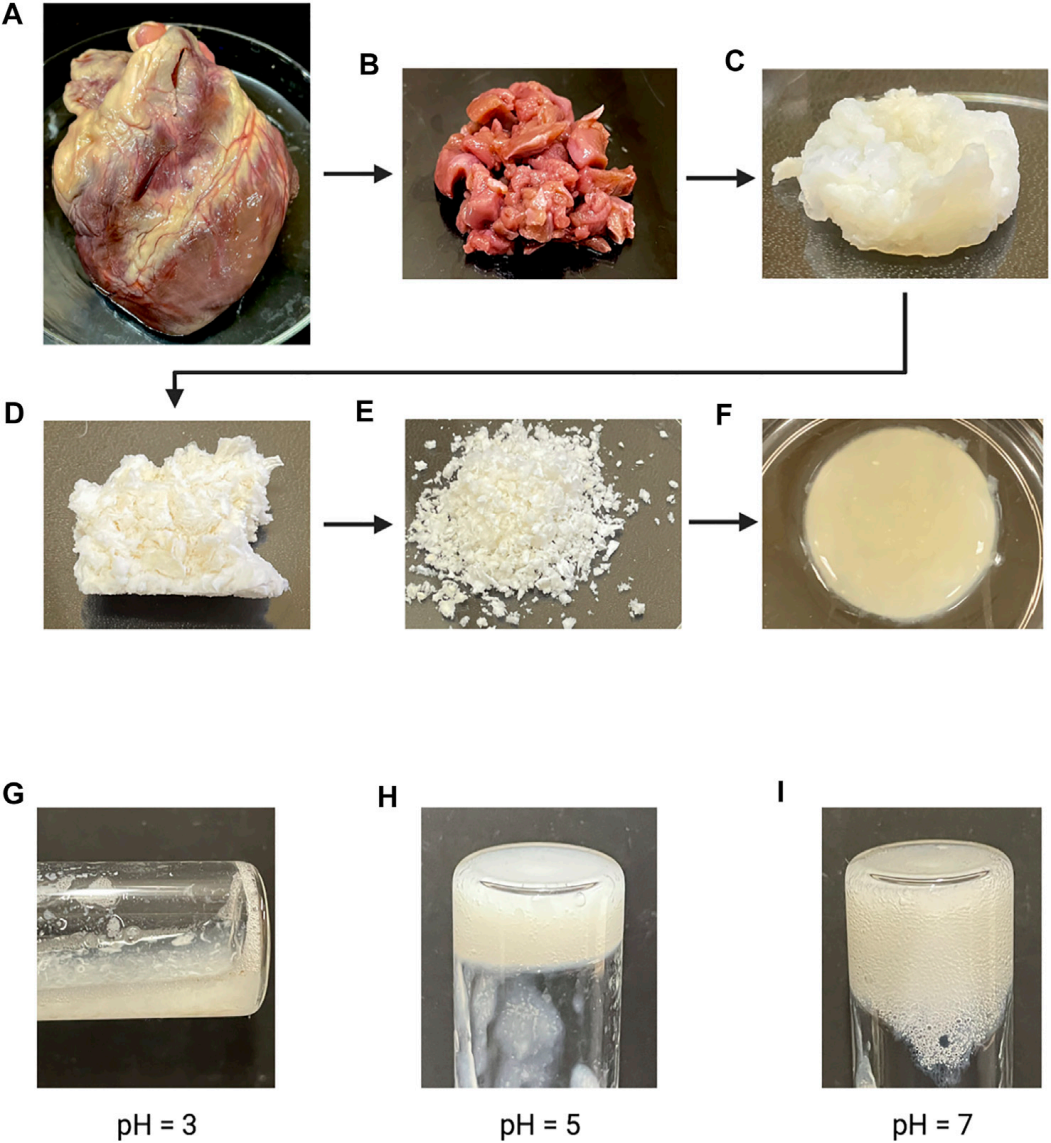
**(A)** Fresh whole porcine heart. **(B)** Cut sections of porcine cardiac tissue prior to decellularization. **(C)** Cut sections of cardiac tissue post chemical decellularization. **(D)** Lyophilized decellularized ECM. **(E)** Powdered decellularized ECM. **(F)** Final gelled form of the enzymatically digested decellularized ECM. **(G–I)** Enzymatically digested decellularized ECM at different pH after a 1 h incubation at 37°C.

**FIGURE 2:**
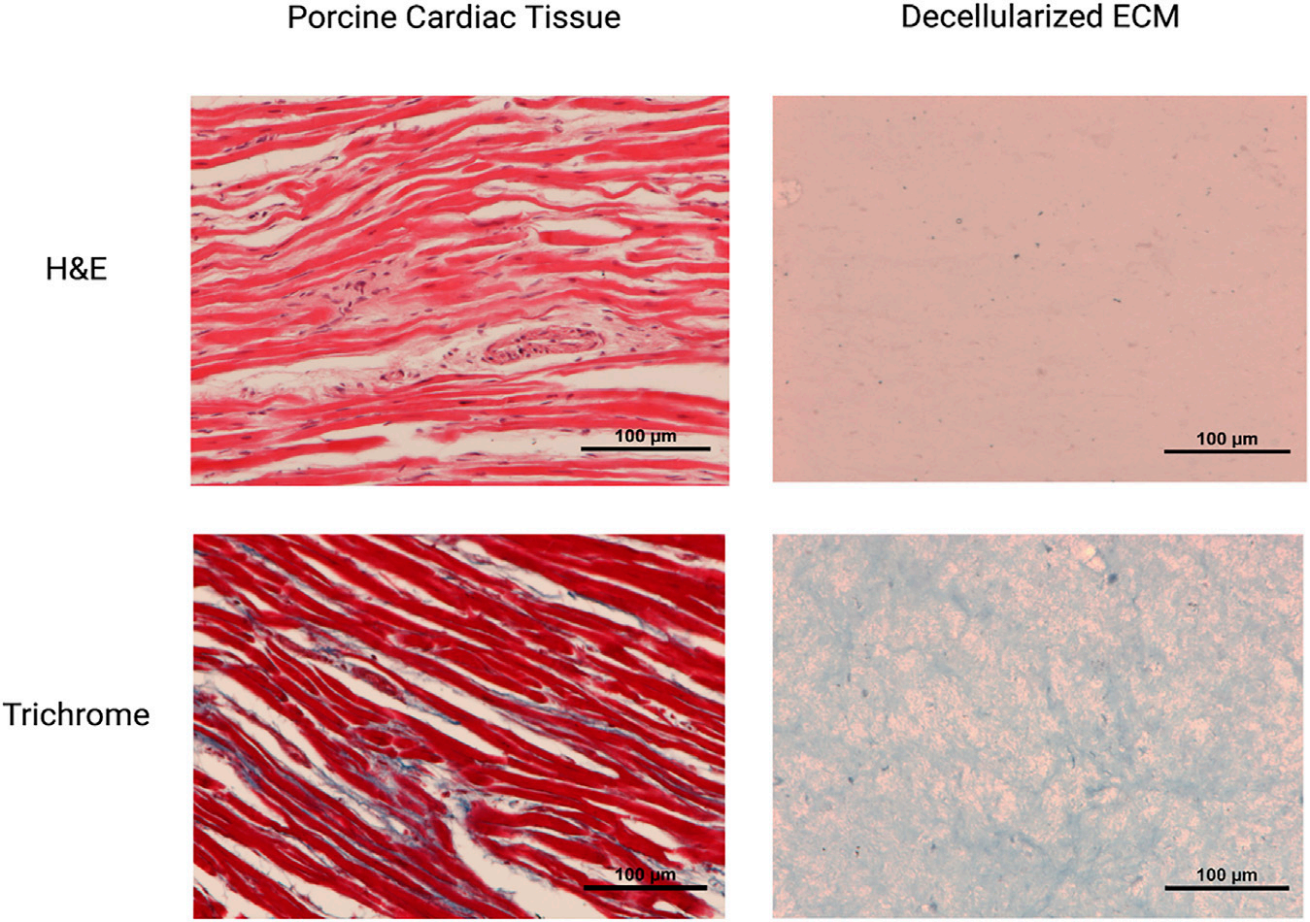
Histological staining of the tissues pre and post decellularization.

**FIGURE 3:**
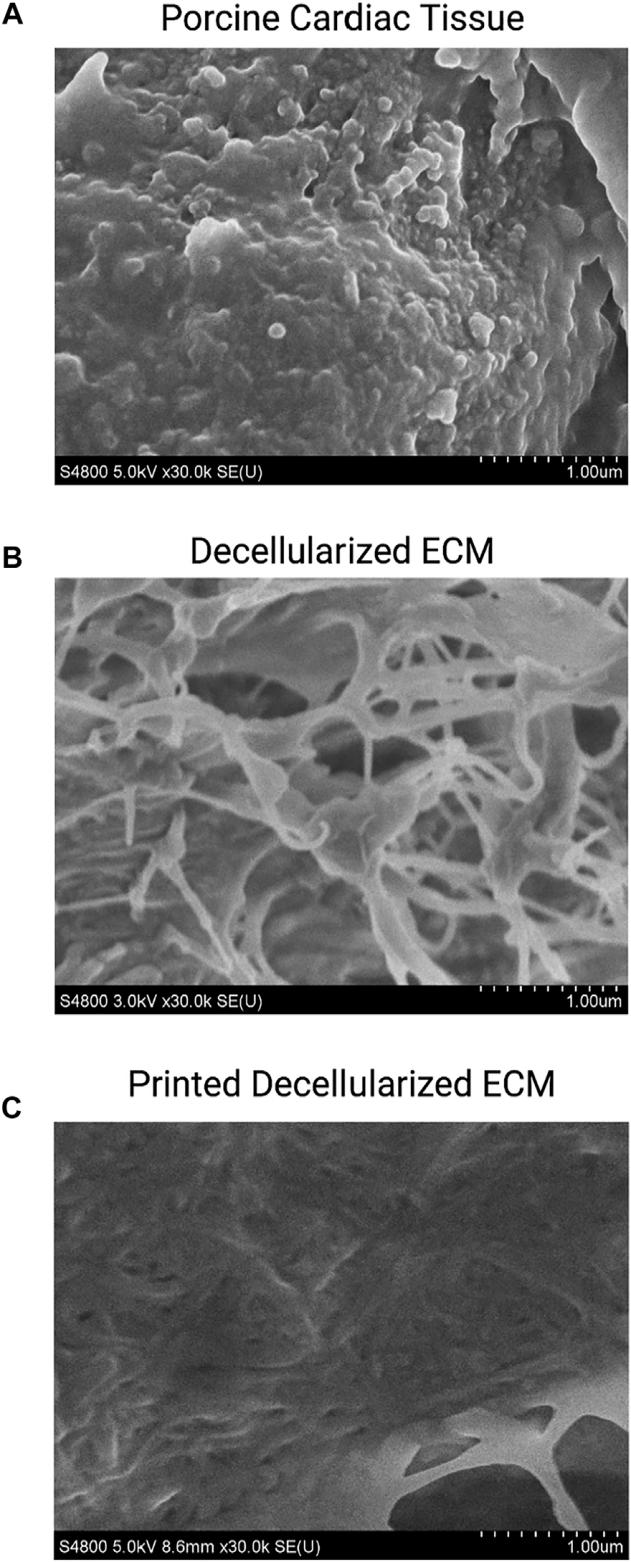
SEM of porcine cardiac tissue **(A)**, decellularized extracellular matrix **(B)**, and printed decellularized matrix **(C)**.

Chemical decellularization of the cardiac tissue resulted in visual, physical changes to the structure and state of the tissue sections (Figures 1A–F). When subjected to temperatures of 37°C post enzymatic digestion the decellularized ECM showed three different stages of crosslinking. At a pH of 3 it was a low viscosity fluid, transitioned to being highly viscous at a pH of 5, and finally crosslinked successfully at a pH of 7 (Figures 1G–I). Hematoxylin and eosin staining of the cardiac tissue pre-decellularization confirmed normal morphology of the cellular structures, no nuclear irregularity or clumping of the chromatin to indicate cancerous areas. Staining with Masson’s Trichrome showed collagenous areas throughout the section; a normal extracellular matrix was visible and necessary for future experiments’ success. Once chemical decellularization, enzymatic digestion, and gelation of the decellularized ECM was completed, the stains were repeated to determine the presence of cellular matter and collagen. Staining showed a lack of cellular structures and nuclear matter; collagen fibrils are uniformly seen throughout the decellularized sample (see Figure 2). Randomly oriented collagen fibrils can be seen in the decellularized ECM’s dense network, whereas the printed decellularized ECM’s topography was similar to that of the porcine cardiac tissue (see Figure 3).

### Mechanical Characteristics

**FIGURE 4:**
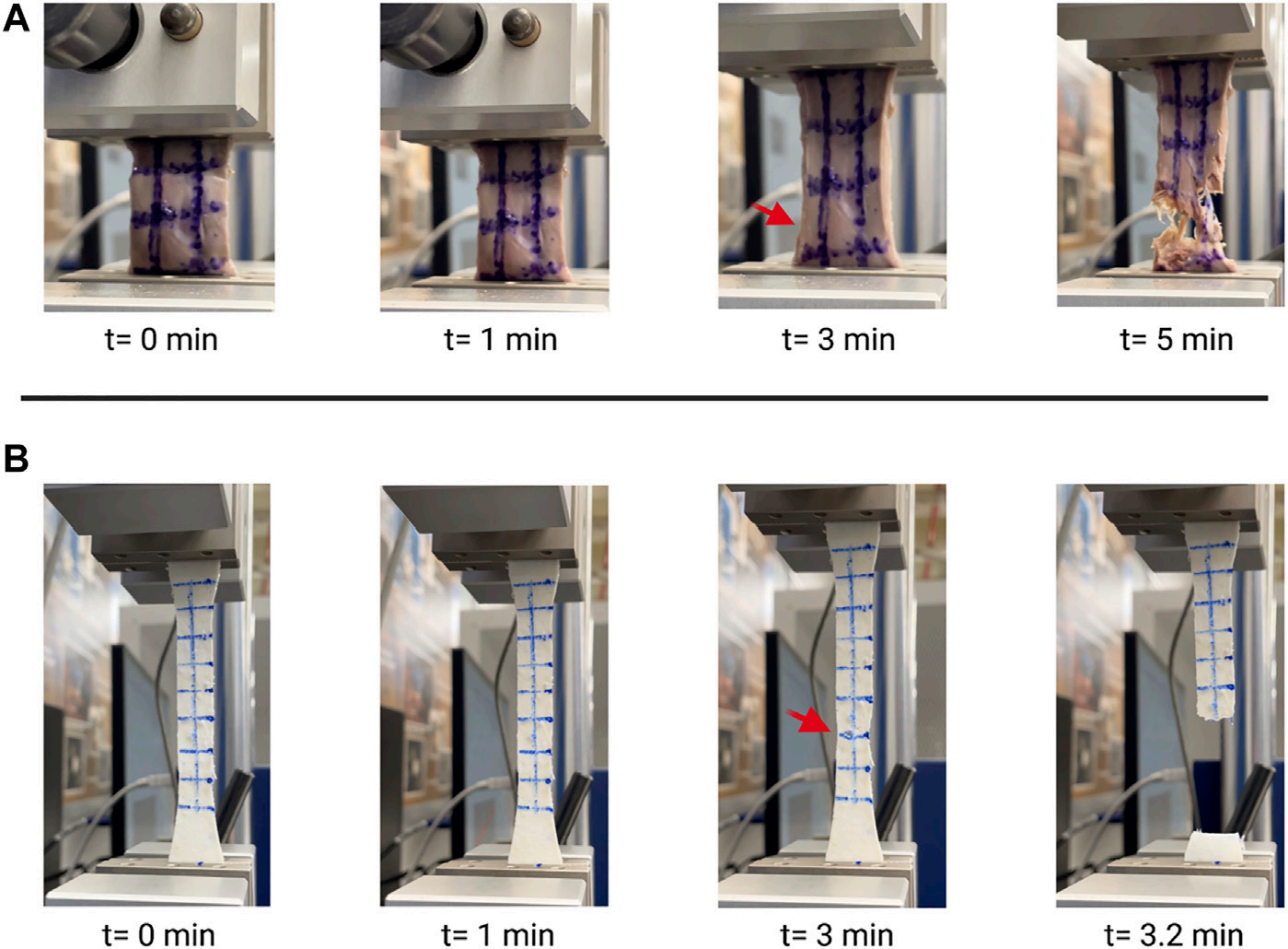
Video stills of porcine cardiac tissue **(A)** and decellularized ECM **(B)** undergoing tensile testing. Red arrows indicate initial failure point.

**FIGURE 5:**
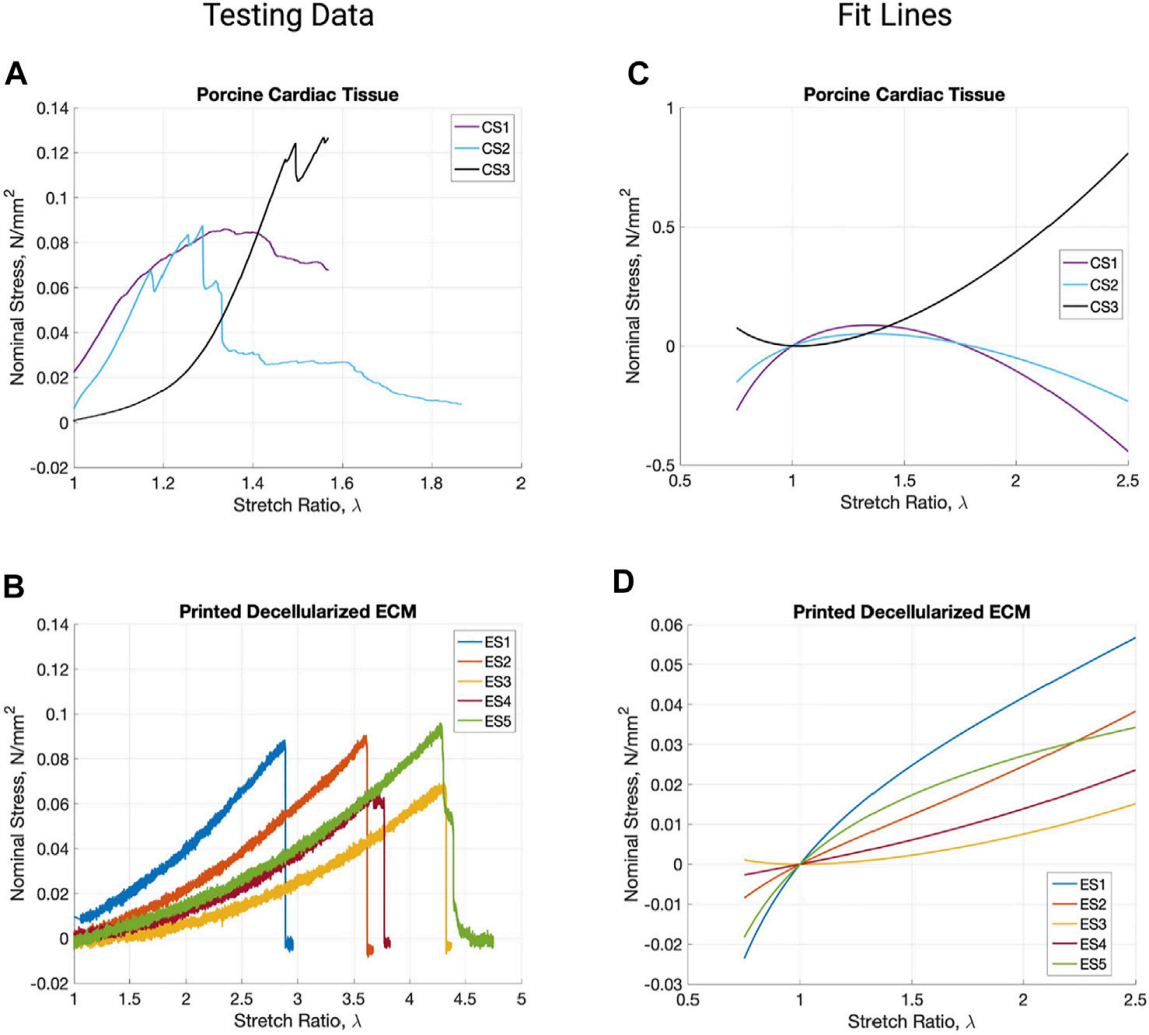
Stress *vs.* stretch ratio graphs of the raw testing data from porcine cardiac tissue **(A)** and printed decellularized ECM **(B)**. Mooney-Revilin fit lines for the testing data curves of cardiac tissue **(C)** and the printed decellularized ECM **(D)**.

Video stills of the tensile testing allowed for precise comparison of the change in length (ΔL) and change in width (ΔH) (Figure 4) in addition to the information gathered for the stress-strain ratio curves. Due to the samples’ large deformations when undergoing forces, a Mooney-Rivlin model was used to find the samples’ effective stresses (Figure 5). The response of the cardiac tissue (Figures 5A, C) indicates an overall higher tensile strength and greater plasticity than that of the printed decellularized ECM (Figures 5B, D). Young’s Modulus and Poisson’s Ratio for the individual samples are shown in Table 1. The printed decellularized ECM (Ē = 920 kPa) exhibited 2.7 times greater elasticity than the porcine cardiac tissue (Ē = 330 kPa). Similarly, the porcine cardiac tissue displayed 1.8 times the deformation $\left(\bar{\nu }=3.93\right)$ prior to failure than the printed decellularized ECM $\left(\bar{\nu }=2.13\right)$ . Neither value showed a significant (*p* < 0.05) difference between the two sample types.

**TABLE 1:** Mechanical Characteristics.

Sample	Young’s modulus (E) kPa	Poisson’s ratio (*v*)
CS1	233	3.17
CS2	288	6.93
CS3	470	1.69
ES1	1,284	1.61
ES2	1,134	0.74
ES3	831	3.00
ES4	576	3.83
ES5	770	1.46

CS1–3 refers to the porcine cardiac tissue samples 1–3. ES1–5 refers to the printed decellularized extracellular matrix samples 1–5.

### Bioprinting

**FIGURE 6:**
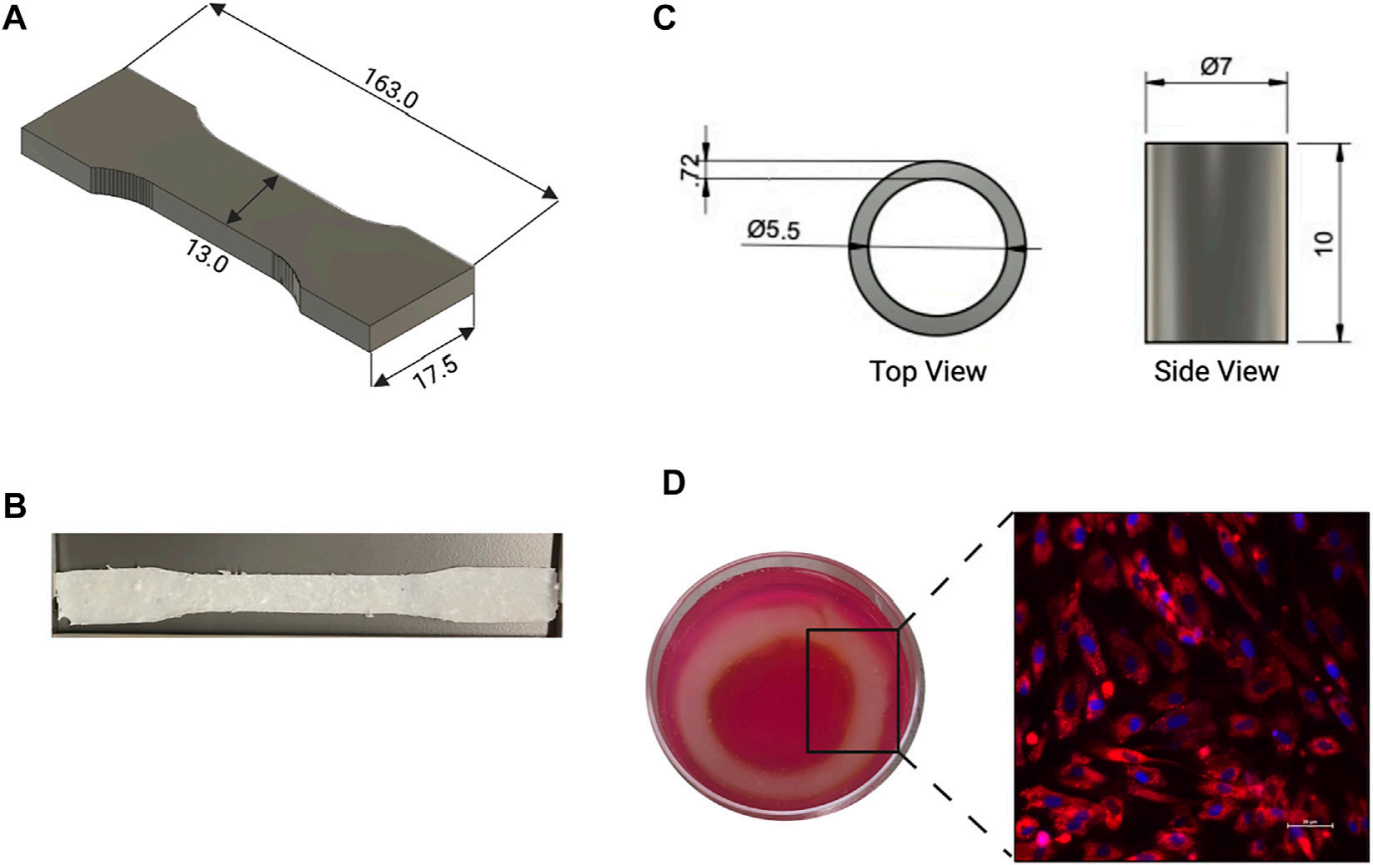
**(A)** CAD and **(B)** image of dog bone shape used for tensile testing of the printed dECM. **(C)** Top and side viewpoint of CAD used to print aortic construct. **(D)** Printed aortic construct in media and confocal image of Human Aortic Smooth Muscle Cells (HASMC) that were grown for 24 h after bioprinting, red represents the membrane (PKH26 general membrane stain), blue represents the nuclear region of the cells (DAPI).

Due to the highly viscous nature of the bioink used a larger gauge needle was selected for bioprinting. The dog bone and cylindrical structures were printed without deformation issues or bleeding of the bioink into the support bath (Figure 6). The printed aortic structures maintained their original shape and dimensions post crosslinking, as did the tensile testing samples. Confocal imaging of the bioprinted HASMC indicates normal cell attachment and morphology 24 h post-print, with no necrotic areas as seen in Figure 6D.

## Discussion

The study examined mechanical characteristics and cellular compatibility of bioprinted decellularized ECM. Successful decellularization is an essential first step in the process; cellular remnants in the bioink cause necrotic pockets, which lead to contamination and complete failure of the construct. Additionally, as the project’s end goal is implantation into a body, rejection is a considerable concern; complete decellularization ensures the possibility of rejection is lowered. Hematoxylin and Eosin staining of the tissues pre-decellularization show normal physiology with clear musculature orientation; once the tissue has undergone decellularization, a drastic change to the physiological properties occurs, with little to no cells present in the sample. Trichrome staining exhibited large quantities of collagen in both samples. Once decellularized, the fibers are no longer uniformly oriented; instead, they are found disbursed throughout the sample, seemingly at random. This can also be seen in the SEM images. Fibrillar collagen and additional elastic fibers comprise approximately 50% of the aorta’s dry weight, making it the primary defining element in the mechanical properties of the large vessel (Harkness et al., 1955; Borst et al., 1983; Jana et al., 2019). Fiber orientation is responsible for the strength of the vessel wall; therefore, the non-uniform orientation of the fibers diminishes the tensile strength of the tissue (Wagenseil and Mecham, 2009).

Similarly, the digestion of the dECM must be carefully monitored. Enzymatic activity is temperature, pH, and concentration sensitive, as is protein structural stability; the lower pH of 3 and 5 lack the environmental factors necessary to promote the electrostatic and hydrophobic interactions between the collagen molecules that facilitate proper thermal crosslinking (Theocharis et al., 2019). At physiological pH (7), not only are the crosslinking capabilities optimized, but the environment for cellular adhesion, proliferation, and signaling is ideal.

Currently, SDS treatment for decellularization, such as what was used, is considered the preferred method for preserving the structural components of elastin and collagen (Grauss et al., 2003; Liao et al., 2007). These previous studies have been done on whole tissue sections and found minor, non-significant differences in the tangent modulus (Zou and Zhang, 2012). Alternatively, bioprinting of the matrix requires more aggressive treatments to the tissue, leading to significant structural and chemical alterations to the matrix. Fresh cardiac tissue samples were compared to the bioprinted samples for uniaxial tensile testing. Though both exhibited hyperplastic properties, the printed dECM samples saw a heightened elasticity, 2.7X that of the cardiac tissue. This elasticity of 920 kPa is 1.8X that of a native infant ascending aorta at normal physiological pressure (60 mmHg) and closer to the elasticity seen under the high stress conditions of higher blood pressure (967.45 kPa) (Ligere et al., 2012).

A lack of stiffness in a vessel is usually associated with structural immaturity, which is remedied by progressive blood pressure increases during fetal development, or aging of the vessel (Dobrin, 1978; Lang et al., 1994; Akira and Yoshiyuki, 2006). In this case, the tested samples contained no cells, thus lacking the additional stability given by the smooth muscle fibers and the biomechanical morphological changes that come from the environmental and biochemical stimuli the cells receive.

Additionally, elasticity of the aortic tissue is greatly affected by surgical interventions. Depending on the type of anastomosis that the physician chooses, the moduli can range from 683.29–1,232.79 kPa for an extended end-to-end anastomosis or 902.39–1,261.35 kPa for an end-to-end anastomosis (Ligere et al., 2012). As the elastic moduli of the dECM falls within the parameters that are seen for post-operative aortic tissues, we anticipate the addition of cells to the construct will bring the overall elasticity down to levels near those of the native aorta.

In a step towards making full aortic constructs, we incorporated human cells by co-printing and culturing ASMC in the dECM. This allowed us to determine cellular viability and adhesion capabilities of the cells within the matrix. 24 h post-printing, the ASMC exhibited normal morphology, and no necrotic areas were visible. The biocompatibility of the dECM post-printing is promising, pointing to the ability of the cells to grow within the matrix for extended periods of time. Overall, the study found an increase in hyperelasticity of the dECM and an ability for ASMC to thrive *in vitro* post-printing, indicating our ability to bioengineer an immature large-scale aortic model, furthering the capabilities of vascular engineering.

## Data Availability

The raw data supporting the conclusion of this article will be made available by the authors, without undue reservation.
